# Immune Thrombocytopenic Purpura, a rare manifestation of active tuberculosis: Case report

**DOI:** 10.1002/ccr3.3031

**Published:** 2020-08-07

**Authors:** Yadhira A. Fajardo, Eliana I. Morales, Luz F. Sua, Liliana Fernández‐Trujillo

**Affiliations:** ^1^ Department of Emergency Medicine Fundación Valle del Lili Cali Colombia; ^2^ Faculty of Health Sciences Universidad Icesi Cali Colombia; ^3^ Department of Internal Medicine, Pulmonology Service Fundación Valle del Lili Cali Colombia; ^4^ Department of Pathology and Laboratory Medicine Fundación Valle del Lili Cali Colombia; ^5^ Department of Internal Medicine, Pulmonology Service, Interventional Pulmonology Fundación Valle del Lili Cali Colombia

**Keywords:** immune thrombocytopenic purpura, immunoglobulin G, steroids, thrombocytopenia, Tuberculosis

## Abstract

Tuberculosis is currently considered a public health issue worldwide. Although a large number of hematological disorders related to tuberculosis have been described, immune thrombocytopenic purpura in patients with active tuberculosis is very rare but must be considered in a patient with no other evident cause.

## Graphical abstract

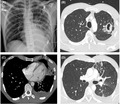

## 1 INTRODUCTION

Although a large number of hematological disorders related to tuberculosis have been described, anemia, leukocytosis, and pancytopenia are the most frequent. Thrombocytopenia is a rare manifestation of active tuberculosis. We present the case of a 38‐year‐old man who presented with subacute respiratory symptoms and a platelet count of 5000 per microliter in the context of active tuberculosis, where no other causes for thrombocytopenia were found.

Tuberculosis (TB) is currently considered a public health issue worldwide and one of the leading causes of morbidity and mortality in Latin America and Colombia.^1^

Thrombocytopenia is a hematological disorder characterized by a platelet count lower than 100,000 per microliter (mcl) and is considered severe with values below 20 000 per mcl. Immune thrombocytopenic purpura (ITP) is a cause of thrombocytopenia that occurs due to antibody‐mediated platelet destruction. It can be primary, or secondary to autoimmune, malignant, or infectious diseases such as systemic lupus erythematosus, lymphoproliferative syndromes, hepatitis C, acquired immunodeficiency virus, or TB.

Although a large number of TB‐related hematological disorders have been described, anemia, leukocytosis, and pancytopenia are the most frequent. Thrombocytopenia is an unusual manifestation of active TB and is usually secondary to nonimmunological disorders, occurring with pancytopenia that develops after bone marrow granulomatous infiltration.^2^ Tuberculosis can potentially infect any organ, but commonly presents as a disease of the lungs which can spread via blood to all organs in the body, such as the pleura, bones, urinary tract, bowels, and skin,. This may cause innumerable manifestations and associated complications, among which ITP is very rare.

## 2 CASE DESCRIPTION

**Figure 1:**
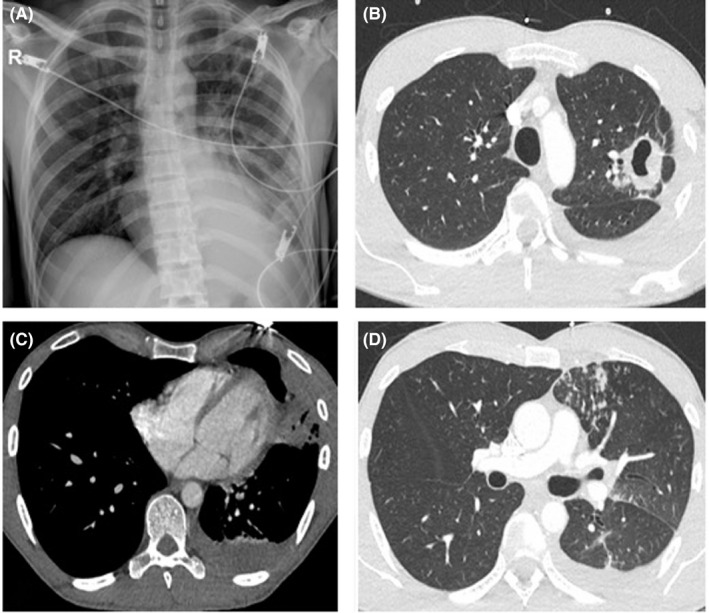
A, Chest X‐ray showing a cavitated lesion with thick edges at the left pulmonary apex associated with infiltrates with alveolar occupation in the left lower lobe. B‐D, Computed tomography showing the left apical cavity with irregular edges with hydro air level associated with inflammatory‐appearing nodules, ipsilateral endobronchial dissemination, and left pleural effusion

A 38‐year‐old man, who was deprived of his liberty at prison for several years until three months before his admission, arrived at another institution for ecchymotic lesions, productive cough, weight loss, anorexia, epistaxis, and low‐grade fever of 20‐day duration, without previous trauma. His past medical history was otherwise unremarkable, and he reported not having previous alterations in clinical and laboratory examinations whatsoever. Laboratory tests at admission were as follows: leukocytes 9,460/mL (NR: 4,230 ‐ 9,070), neutrophils 82% (NR: 34‐67.9), hemoglobin 10.4 g/dL (NR: 13.7‐17.5) hematocrit 30 mg/dL (NR: 40.1‐51), erythrocyte sedimentation rate 83.3 mm/h (NR: 0‐15), platelets 5000/mL (NR: 163 000‐337 000), PT 11.8 (control: 11.3), PTT 29 (control: 30.9), Fibrinogen 509 mg/dL (NR: 150‐400), D‐dimer 13 080 μg/mL (NR: 0‐0.5), total bilirubin 0.95 (NR: 0‐1.2), lactic dehydrogenase (LDH) 206 IU/L (NR: 135 ‐ 225), alkaline phosphatase 80 IU/L (NR: 20‐140), folic acid 11.49 ng/mL (2.6‐17), and vitamin B12 357 ng/mL (NR: 197‐771). He had negative ELISA for HIV, negative hepatitis B surface antigen, and hepatitis C antibody; nonreactive RPR, direct bacilloscopy was negative; urinalysis had macroscopic hematuria; and urine culture was negative. A chest X‐ray showed a cavitating lung lesion with thick edges in the left pulmonary apex (Figure 1A). The case was interpreted as an idiopathic thrombocytopenic purpura, and due to platelet count, intravenous (IV) immunoglobulin G was started, along with methylprednisolone 1 mg/kg/day. Ivermectin 70 drops single dose and Albendazole 400 mg/d for 3 days were initiated before immune therapy, in order to prevent opportunistic helminthic infections.

On his fourth day of hospitalization, he developed hemodynamic instability, given by persistence of active bleeding: hematemesis, epistaxis, hematuria, and severe secondary anemia, requiring blood transfusion and apheresis platelets. He was referred to our institution in poor general conditions. On physical examination: blood pressure 110/70 mm Hg, heart rate 75 bpm, respiratory rate 13 bpm, oral cavity with petechial lesions, coarse crackles in both lungs, multiple petechiae, and ecchymoses spread all along the extremities. The rest of the physical examination was unremarkable. Laboratory tests showed leukocytes 11 200/μL, neutrophils 10 240/μL, lymphocytes 530/μL, hemoglobin 9.8 g/dL, hematocrit 28%, platelets 1000/μL, PT 15, INR 1.38, PTT 27.5, Fibrinogen 738, total bilirubin 0.95, ALT 12 mg/dL (NR: 0‐41), AST 28 mg/dL (NR: 0‐38), LDH 397 IU/L, IgA: 1.88 (NR: 0.7‐4.0), IgG: 33 (NR: 7‐16), and IgM: 1.17 (NR: 0.4‐2.3). HIV antibodies were negative, and viral load was undetectable. Chest X‐ray showed a thick‐walled cavitating image in the left pulmonary apex with effacement of the left costophrenic angle probably due to effusion or pleural thickening. A thoracic CT scan showed a left apical cavity with irregular borders and hydro‐aerial level, with nodules of inflammatory aspect, ipsilateral endobronchial dissemination, and left pleural effusion (Figure 1B‐D). Bacilloscopy was positive in urine. Bone marrow aspirate and biopsy were negative for neoplastic infiltration. A fibrobronchoscopy plus bronchoalveolar lavage (BAL) was performed, showing generalized severe endobronchial inflammation without obstruction or masses. BAL showed inflammatory cells in cytology study, macrophages, and plentiful lymphocytes with no evidence of malignancy. Ziehl‐Neelsen stain was positive +++, and galactomannan was negative. GeneXpert was positive without evidence of antibiotic resistance. The aforementioned test is a rapid, simple‐to‐use nucleic acid amplification test, which detects DNA sequences specific for M. tuberculosis and rifampicin resistance using polymerase chain reaction.

Supervised shortened oral anti‐TB treatment was initiated on a weight‐based dosing, as follows: rifampicin 150 mg, isoniazid 75 mg, pyrazinamide 400 mg, ethambutol 275 mg four tablets, and pyridoxine 50mg daily. Due to thrombocytopenia and evident hemorrhagic manifestations, he required multiple blood transfusions and apheresis of platelets. Following the findings in the bone marrow in which there was no neoplastic or lymphoproliferative infiltration, a diagnosis of ITP was made in the context of active tuberculosis. Concomitant treatment with dexamethasone 40 mg/d for 4 days and immunoglobulin G 50 g/d for 2 days was initiated.

The patient improved his general condition one week after starting the therapeutic scheme, without new bleeding episodes and improvement of laboratory tests: leukocytes 5190/μL, neutrophils 3160/μL, lymphocytes 1170/μL, hemoglobin 10.6, hematocrit 30.8%, platelets 53 000/μL, creatinine 0.77 (NR: 0.67‐1.17), ALT 64.4, AST 73.2, sodium 132 mmol/L (NR: 136‐145), potassium 4.49 mmol/L (NR: 3.5‐5.1); chloride 98 mmol/L (NR: 98‐107), magnesium 2.19 mmol/L (NR: 1.59‐2.56), and phosphorus 3.34 mmol/L (NR: 2.5‐4.5).

The patient continued steroid therapy with oral prednisolone 1 mg/kg with a gradual dose decrease in the following month as well as supervised shortened TB treatment for six months, without adverse effects or recurrence of ITP.

## 3 DISCUSSION

TB is a common disease in prisons in developing countries, due to risk factors such as overcrowding, poverty, immunodeficiency due to other diseases, among others. Our patient was exposed to these for years prior to hospitalization.^1^

ITP is an autoimmune disease characterized by thrombocytopenia as the only hematological manifestation. The two main diagnostic criteria for ITP are thrombocytopenia in the context of a normal blood count, a normal smear, and the exclusion of primary conditions capable of causing thrombocytopenia. There are two forms of ITP: the infantile acute variety, mostly postviral and self‐limited; and adult ITP that tends to be chronic, more common in women between the second and fourth decades of life.^3^ Many infectious and noninfectious diseases can trigger secondary ITP.

In patients with TB, ITP is very rare. There are only a few cases. TB‐induced thrombocytopenia is more commonly caused by nonimmunological mechanisms and typically manifests in the context of pancytopenia that develops secondarily to granulomatous bone marrow infiltration. The association between TB and ITP is extremely rare.^2^, ^4^, ^5^, ^6^, ^7^ TB most commonly presents with hematological alterations such as anemia, leukocytosis, leukopenia, or pancytopenia.^8^, ^9^

The pathophysiology of isolated thrombocytopenia in tuberculosis is not well known. A proposed hypothesis describes an immune destruction of platelets through circulating antiplatelet antibodies, which are probably produced by an abnormal stimulation of B lymphocytes by *Mycobacterium tuberculosis*.^5^, ^6^, ^7^, ^9^

Other causes of thrombocytopenia, besides mycobacterial bone marrow infiltration, are histiophagocytosis, thrombotic thrombocytopenic purpura, disseminated intravascular coagulation, the immune type (present in this case), and the secondary to adverse effects of anti‐TB drugs, especially rifampicin and isoniazid.^7^ Bleeding is the most common manifestation of ITP, especially of mucosae and gastrointestinal tract.

The diagnosis of TB in this case was initially based on clinical and radiological findings on the CT scan; subsequently, urine and BAL samples were positive for mycobacterial ZN‐staining, which allowed diagnosis confirmation.

The first line of treatment for ITP includes IV steroids, IV immunoglobulin G, and anti‐D immunoglobulin. The use of high‐dose methylprednisolone is recommended for three to five days followed by oral maintenance therapy with gradual dose reduction completing four to eight weeks depending on platelet count follow‐up. Also, adding IV immunoglobulin G 0.4 g/kg/d for 5 days can achieve a greater and faster response in approximately 80% of the cases.

The therapeutic response exhibited as a progressive platelet count increase with steroids and IV immunoglobulin G, along with a confirmed TB diagnosis, altogether supported the diagnosis of ITP secondary to TB. Some authors have reported on the therapeutic use of steroids plus anti‐TB treatment, which makes it very likely that this blood dyscrasia actually represents an actual unusual manifestation of TB.^8^, ^9^

Clinically, it is known that steroids improve purpuric bleeding before the platelet count actually increases. The early effect is due to the decrease in vascular permeability, while the late effect involves immunoglobulin G. A blockade of Fc receptors in reticuloendothelial cells and suppression of the production of antibodies and their binding is suggested as a possible mechanism that may be the result of anti‐idiotype antibodies that bind to antiplatelet antibodies which modulate the immune response.^4^

In this case, methylprednisolone plus immunoglobulin G were administered, achieving significant improvement as seen in the increase on platelet count.^3^, ^10^ Steroids as a cornerstone of treatment are continued orally with gradual dose decrease to complete 4‐8 weeks, always keeping in mind that the underlying cause must be treated. In this case, TB was treated with supervised shortened regimen, achieving a complete remission of both TB and ITP.

In conclusion, TB continues to be a public health issue worldwide, especially among developing countries vulnerable populations. Comorbidities such as diabetes mellitus, chronic kidney disease, and immunosuppression can favor TB infection. Diagnosis and treatment may represent a challenge, due to its multiple manifestations, in this case, a rare hematological alteration in the presence of active infectious disease.

## CONFLICT OF INTEREST

None declared.

## AUTHOR CONTRIBUTIONS

All authors contributed to data analysis, drafting and revising the article, gave final approval of the version to be published, and agreed to be accountable for all aspects of the work. YAF and LFT: conceptualized and designed, reviewed the literature, wrote and corrected the manuscript, and involved in final approval of manuscript. EIM and LFS: conceptualized and designed, reviewed the literature, and involved in final approval of manuscript.

## ETHICS APPROVAL AND CONSENT TO PARTICIPATE

This report was prepared in accordance with the ethical standards of the institutional ethics committee and with the 1964 Helsinki Declaration. We have approval letter of Ethics Committee in biomedical research IRB/EC No. 255‐2019 of the Fundación Valle del Lili to publish this manuscript.

## CONSENT FOR PUBLICATION

Written informed consent was obtained from the patient for publication of this case report and any accompanying images. A copy of the written consent is available for review by the editor‐in‐chief of this journal.

## Data Availability

All data and material are available for sharing if needed.
